# Development and Implementation of a New Telerehabilitation System for Audiovisual Stimulation Training in Hemianopia

**DOI:** 10.3389/fneur.2017.00621

**Published:** 2017-11-21

**Authors:** Francesca Tinelli, Giovanni Cioni, Giulia Purpura

**Affiliations:** ^1^Department of Developmental Neuroscience, IRCCS Stella Maris Foundation, Pisa, Italy; ^2^Department of Clinical and Experimental Medicine, University of Pisa, Pisa, Italy

**Keywords:** telerehabilitation, audiovisual stimulation, visual field defects, hemianopia, children

## Abstract

Telerehabilitation, defined as the method by which communication technologies are used to provide remote rehabilitation, although still underused, could be as efficient and effective as the conventional clinical rehabilitation practices. In the literature, there are descriptions of the use of telerehabilitation in adult patients with various diseases, whereas it is seldom used in clinical practice with child and adolescent patients. We have developed a new audiovisual telerehabilitation (AVT) system, based on the multisensory capabilities of the human brain, to provide a new tool for adults and children with visual field defects in order to improve ocular movements toward the blind hemifield. The apparatus consists of a semicircular structure in which visual and acoustic stimuli are positioned. A camera is integrated into the mechanical structure in the center of the panel to control eye and head movements. Patients can use this training system with a customized software on a tablet. From hospital, the therapist has complete control over the training process, and the results of the training sessions are automatically available within a few minutes on the hospital website. In this paper, we report the AVT system protocol and the preliminary results on its use by three adult patients. All three showed improvements in visual detection abilities with long-term effects. In the future, we will test this apparatus with children and their families. Since interventions for impairments in the visual field have a substantial cost for individuals and for the welfare system, we expect that our research could have a profound socio-economic impact avoiding prolonged and intensive hospital stays.

## Introduction

Telerehabilitation, defined as the method by which communication technologies are used to provide remote rehabilitation, although still underused, could be as efficient and effective as the conventional clinical rehabilitation practices (1). In the literature, we can find some descriptions of the use of telerehabilitation in adult patients for various types of disorder, whereas it is seldom used in clinical practice with children and adolescents (2).

The development and use of telerehabilitation program are slow because they are affected by many logistical factors, such as regional economic resources, medical technical support systems, and population quality, but their potential is very high, as they are conceived and studied to improve patients’ ability to perform activities from daily life, thereby increasing their independence (3). For example, for adult post-stroke patients, telerehabilitation is widely used with the main goal of giving disabled people the same quality of motor, cognitive, and neuropsychological rehabilitation at home as they would have in-home visit and day-care rehabilitation (4, 5–7).

So far, the application of telerehabilitation during childhood has been primarily limited to preterm babies (8) and children with hemiplegia (9, 10), with autism spectrum disorders (11), with speech and language disorders (12, 13), and with learning difficulties (14–16). Despite the well-known impact of visual defects on cognitive functioning and neurological recovery (17), no study has yet investigated the application of telerehabilitation with children with visual impairments.

Here, we describe an innovative telerehabilitation platform, which consists in an audiovisual telerehabilitation (AVT) system, developed for children and adults with visual field defects caused by post-chiasmatic brain lesions. The AVT system allows patients to exercise independently, in an intensive, active, and functional way and in a familiar environment, under remote supervision; it consists of a mobile device platform with remote control, which is accessible directly from home and suitable both for adults, adolescents, and children from the age of 8.

The AVT system is based on a very promising multisensory audiovisual therapy, originally developed for the treatment of adults and children with visual field defects caused by brain lesions (18, 19). Basically, this training aims to stimulate multisensory integration mechanisms in order to reinforce visual and spatial compensatory functions (i.e., implementation of oculomotor strategies). In this first phase of the study, we tested the feasibility and efficacy of AVT in three adult patients with chronic visual field defects, in order to explore how the apparatus can be implemented at home.

## Subjects

The first patient (S1) was an adult male, affected by a cerebral stroke at 71 years of age. The stroke caused a lesion in the left occipital cortex and consequently a right hemianopia and a slight right hemiplegia, but during the first 3 months after the stroke the patient gradually showed a spontaneous motor and visual recovery. The patient was assessed for the first time in this study at 1 year after the stroke and showed a persistence of an inferior right quadrantanopia.

The second patient (S2) was a young adult with drug-resistant epilepsy caused by a focal cortical dysplasia type 2a in the left temporal–occipital region, treated with a left temporal–occipital lobectomy at the age of 22 years. After surgery, the patient had a complete right homonymous hemianopia.

The third patient (S3) was a young man 40 years old who needed surgery for a meningioma in right hemisphere. After neurosurgery, the subject reported a partial left homonymous hemianopia.

**Figure 1:**
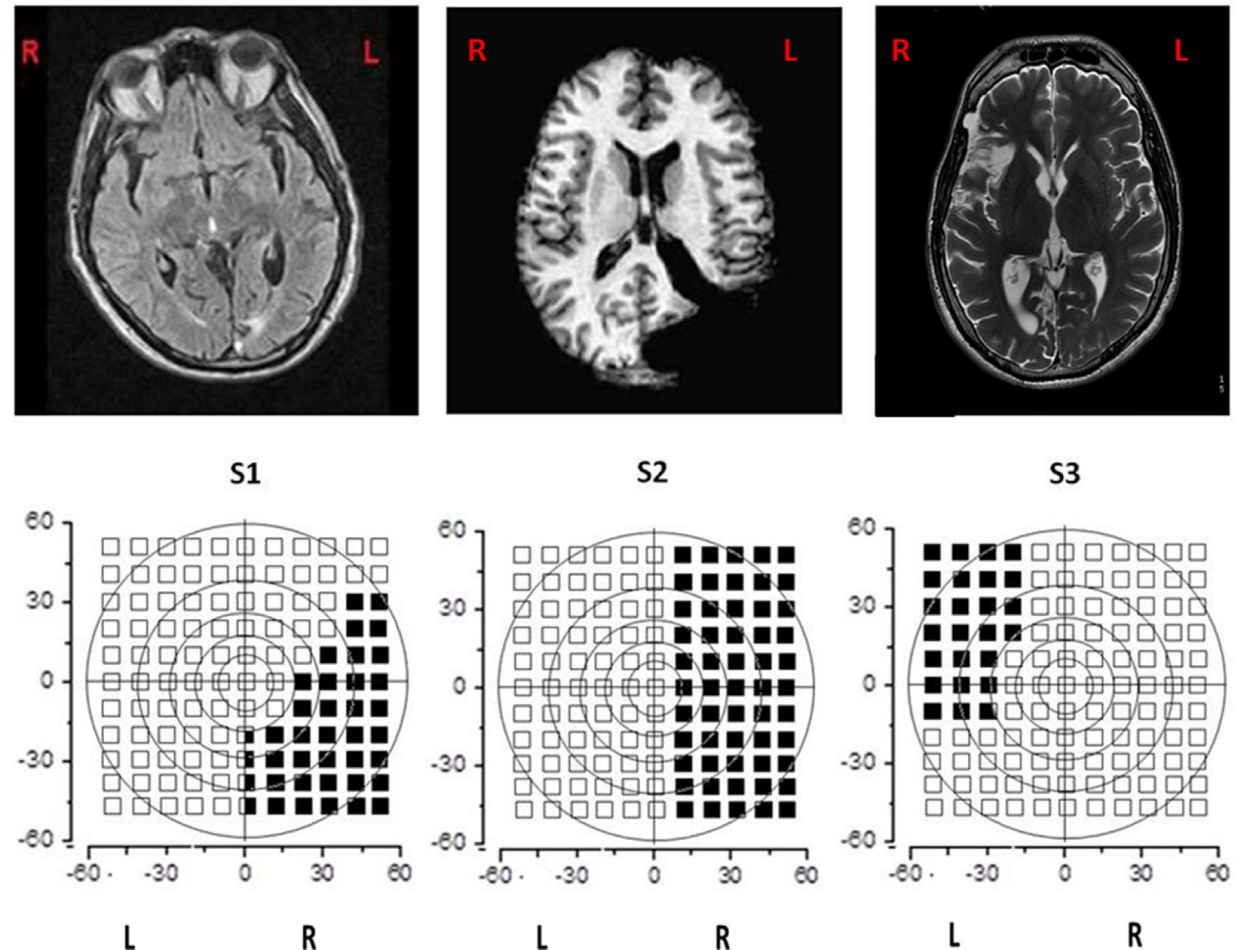
Magnetic resonance imaging of the brain and visual field campimetry of S1, S2, and S3.

Figure 1 shows MRI and visual field campimetry of all three patients. All the subjects were recruited for this experimental training more than 1 year after lesion onset, so that spontaneous recovery was concluded and none of them had undergone other types of rehabilitation specific for hemianopia.

The study was carried out in accordance with the recommendations of the Scientific Institute “Stella Maris Foundation” institutional review board. The project study was reviewed and approved by ethics committee of Scientific Institute “Stella Maris Foundation.” Patients signed an Informed Consent Form, in accordance with the Declaration of Helsinki, authorizing the storage and study of their clinical material for research aims.

## Materials and Equipment

The apparatus consists of a semicircular structure, in which the visual and acoustic stimuli are positioned. It is a plastic arch-shaped device, fixed horizontally on the table surface. The arch can be rolled up and kept in its tube-container and is easily transportable.

Compared with previous audiovisual stimulation instruments (18–20), the height and length of the apparatus are greater because there are two horizontal rows of visual stimuli (LEDs) for a total length of 192 cm, height of 32 cm and thickness of 1.2 cm. The length depends on the extension of the visual field that we want to cover: with this instrument we can cover a range of 180°—the equivalent of the entire visual field. This is an important key factor because it allows us to rehabilitate not only complete hemianopic defects but also partial visual field defects. The height depends on the fact that we have two different fixation points, which are located in the center of the instrument and on the same lines as the LEDs, so that we can use the instrument for the rehabilitation of hemianopia but also for patients with upper or lower quadrantanopia, simply choosing the lowest or the highest fixation point. The thickness depends on the fact that the instrument must be transportable, permitting use at home.

**Figure 2:**
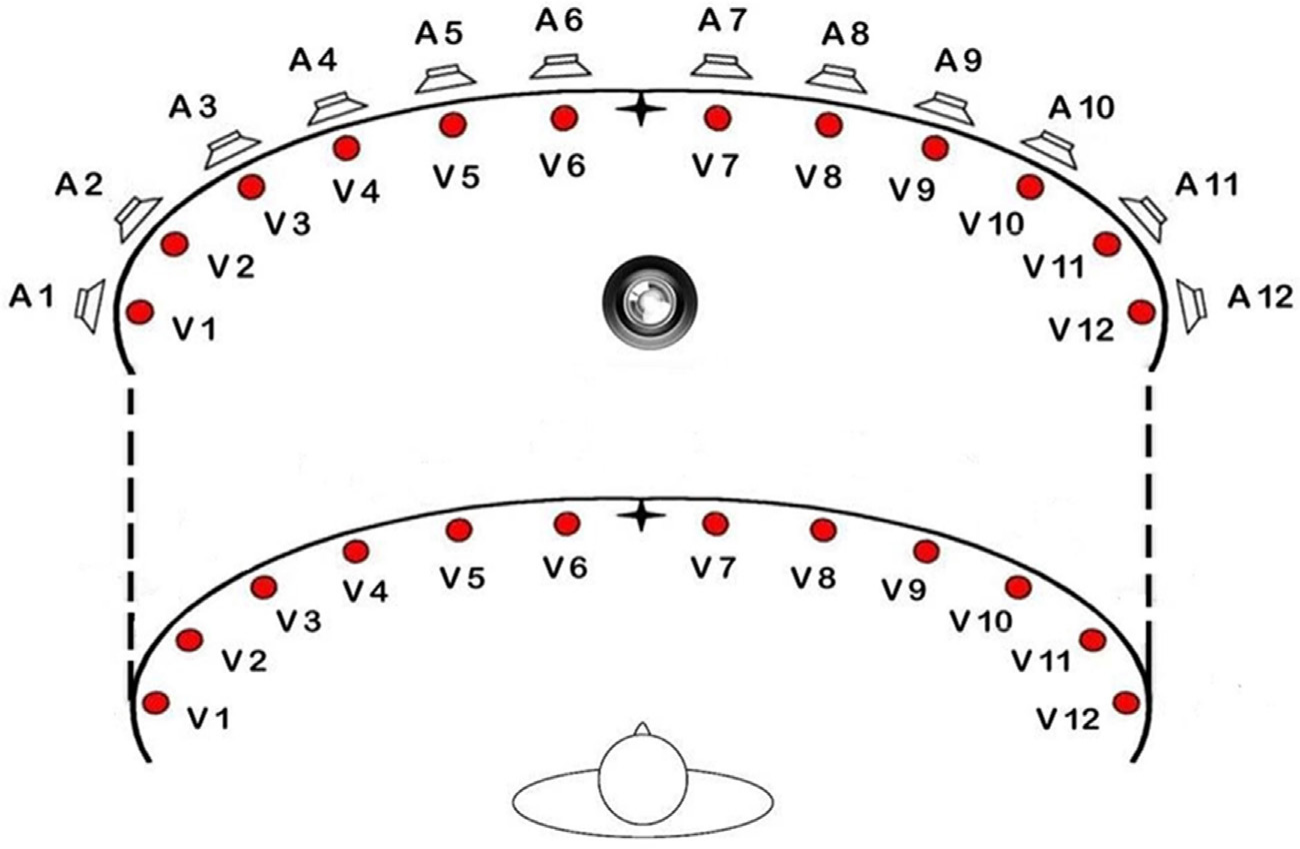
Schematic view of the position of loudspeakers and light displays of the training apparatus.

The LEDs for the visual stimuli have a diameter of 0.5 cm. For every row, there are 12 visual stimuli (for a total of 24 LEDs), at an eccentricity of 8, 24, 40, 56, 72, 88° from the central fixation point in the left and in the right hemifield (from V1 = 88° left to V12 = 88° right). The LEDs are able to perform the full range of possible colors but for this experiment we used red, as in previous studies. At the same degrees of eccentricity, 12 acoustic stimuli (piezoelectric loudspeakers) are positioned (from A1 = 88° left to A12 = 88° right). The acoustic stimuli emitted by the loudspeakers consist in a sound with a frequency of 2,800 Hz (see Figure 2).

A camera is integrated into the mechanical structure in the center of the panel between the two fixation points. Before the start of the trial, the camera performs an initial calibration of the position of the patient’s head and eyes. The camera works even in a condition of low luminosity. The system is programmed to suspend the administration of stimuli if the patient does not maintain the point of central fixation during the inter-trial interval or if the patient moves his head. To facilitate the calibration and the repositioning of the head and gaze between stimuli and during sessions, but also to maintain maximum control of the correct position during the telerehabilitation session, 4 LEDs in the shape of an arrow may flash to suggest how the patient should move his head to find the correct fixation point.

**Figure 3:**
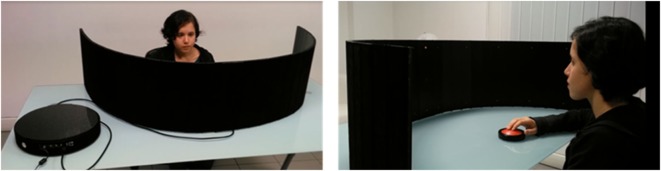
Hardware of training apparatus.

The apparatus is equipped with a circular base where the hardware is located. A large circular red button, connected *via* wireless to the software, is used by the patient to report the detection of the visual stimuli (see Figure 3). The patient can decide when to start the session by pressing the button for 3 s. A flashing light signals the beginning and end of each session.

The patient sits on a chair facing the apparatus at a distance of 57 cm (at this distance 1 cm corresponds to 1° and for this reason we are sure that the instrument covers 180°), looking straight ahead, with his/her body midline aligned with the center of the apparatus, in a dimly lit and sound-attenuated room.

The instrument is installed and initialized at the participant’s home; this involves checking that the system is fully operational and providing the patient and a family carer with instructions regarding its usage. Patients are given a telephone number for technical support or assistance. The patients’ user interface, managed *via* tablet, allows them to select the training session, review their performance history, and send daily scores to the therapist. The level of difficulty of the training schedule is set daily by the therapist, who can adjust the training program remotely, according to individual progress. If no or low use is noticed, the therapist can send a motivational message. Before and after the training, patients undergo a clinical assessment at the hospital labs, conducted by an evaluator blind to the treatment procedure.

The software is very flexible. Lights and sounds can be easily activated in every possible position, color and intensity can be varied by the therapists or patients. These new features were designed to permit the use of the instrument for different types of pathologies.

## Procedures

This telerehabilitation project, specifically designed for patients with visual field defects, is based on a previously developed audiovisual compensatory training. In fact, behavioral studies in humans have shown that audio–visual interaction can improve visual detection (21, 22) and visual localization (23, 24), and reduce saccadic reaction times (25–29). In particular, it has been demonstrated that a sound, spatially and temporally coincident with a visual stimulus, can improve visual perception in the blind hemifield of hemianopic patients (30, 31). This facilitatory effect probably involves the activation of multisensory neurons in the Superior Colliculus (31, 32).

### Training Procedure

After the pre-treatment testing procedure (see the section “Assessment Procedure”), the apparatus was delivered to the patient’s home. The audiovisual Training was administered by the patient and performed at home at least 5 days a week, following the therapist’s indications. The duration of daily sessions varied according to the fatigability of patients.

Within the rehabilitation sessions, three types of stimuli were delivered: (1) unimodal visual condition (presentation of only visual stimuli); (2) unimodal acoustic condition (presentation of only acoustic stimuli); (3) bimodal audiovisual condition (presentation of visual stimuli accompanied by sounds). In the Bimodal audiovisual condition, the sound could be in the same spatial position as the visual stimulus (spatially congruent), or it could appear with a nasal/temporal difference of 16° or 32° (spatially incongruent).

The patients were required to look at the central fixation point and then shift their gaze toward the visual stimulus, without head movements; whenever the visual target was seen, its detection was reported by pressing the response button. The presentation of the stimulus occurred only if the subject was looking at the central fixation point, as detected by the camera. The patient had to detect the presence of the visual stimulus by pressing a button and was asked to ignore the presence of acoustic stimuli. If the patient does not keep his head and gaze on the central fixation point (as in the initial calibration), the system does not permit the administration of the stimuli and the session goes into standby mode.

The intensity and type of the stimulation of the visual field varied according to the site of the visual field defect. For instance, in the case of left-sided hemianopia, a more intense stimulation of the left hemifield was given, comprising unimodal visual stimuli, unimodal acoustic stimuli, and spatially congruent audiovisual stimuli, which were presented in the spatial positions of the left hemifield with a probability of 2% (from 1 to 5 positions). Audiovisual spatially incongruent stimuli were presented in the hemianopic field with a probability of 3% each (V1 with a nasal disparity of 16° and 32°, V2 with a temporal disparity of 16° or with a nasal disparity of 16° and 32°, V3 and V4 with a temporal disparity of 16° and 32° or with a nasal disparity of 16° and 32°, V5 with a temporal disparity of 16° and 32°). On the intact side (right hemifield), unimodal visual stimuli, unimodal acoustic stimuli, and spatially congruent audiovisual stimuli appeared with a probability of 1% (from 8 to 12 positions). In this hemifield, audiovisual spatially incongruent stimuli were presented only from the V9, V10, and V11 positions with a nasal disparity of 32°, with a probability of 3% each. Catch Trials were also included (no acoustic stimulus or visual stimulus) with a probability of 1%. In the case of right hemianopia, the situation was reversed. The different conditions of stimuli were presented in a random order. Each block consisted of 100 visual stimuli. The visual stimuli and acoustic targets had the same 100-ms duration. The interstimulus interval (ISI) ranged randomly between 2,000 and 4,000 ms.

Since visual exploration in hemianopic patients is usually difficult and time-consuming, the training was simplified by having different temporal intervals between the two stimuli. In this way, the therapist can guide the patient better during the rehabilitation program and can control the progressive improvement of his visual exploration skills. This method has already been described in the literature (18, 19). In accordance with this procedure, the rehabilitation program was divided into six sessions, based on the principle of stimulus onset asynchrony (SOA), namely, the time distance between the acoustic stimulus and visual target. Treatment started with 500 ms of SOA, i.e., the auditory stimulus preceded the visual target by 500 ms, and SOA was reduced in steps of 100 ms (i.e., 400, 300, 200, and 100 ms) up to the last session of training, in which stimuli were simultaneous (i.e., 0 ms of SOA). Each SOA session ended when a hit ratio of at least 50% in the unimodal visual condition was obtained in the blind hemifield. This was computed by averaging all the executed blocks of the day at each specific SOA. Once the subject reached this criterion, the next SOA session began. Treatment ended when subjects detected more than 50% of the unimodal visual stimuli for three consecutive blocks of trials in the simultaneous presentation of audiovisual stimuli (last SOA session). This percentage, despite representing a low performance level, was positively correlated with visual search amelioration in an adult pilot experiment (18, 33) and in our pilot study with children (19), aimed at establishing a criterion for the end of treatment. For this reason, the duration of the training program depends on the results reported by the subject on a daily basis and usually lasts from 4 to 6 weeks.

### Assessment Procedure

Treatment efficacy was evaluated by testing all the patients according to a multiple design. Each patient was tested before the treatment, immediately after the treatment and after a resting period (follow-up). The baseline assessment and the post-training assessment were performed immediately before and after the training sessions (baseline assessment: 1–5 days before the start of the training program; post-training: 1–5 days after the end of the training program). Follow-up was performed 6 months after the end of treatment for S1, while for S2 it was performed after 12 months, and for S3, after 9 months. The follow-up was done at a different time from the end of the treatment to demonstrate the long-term effect of the training intervention. For the assessment, we used two visual detection tests, which were performed with the training apparatus in the hospital (Unimodal Visual Test and Bimodal Audiovisual Test). Additionally, all participants underwent a computerized visual field perimetry (KOWA AP 340 system) before and after the training.

Before the beginning of the training procedure we made sure that none of the subjects had hearing problems by using an Unimodal Acoustic Test.

### Unimodal Visual Test

During the Unimodal Visual Test only visual stimuli were presented, without sounds, in order to assess the ability to detect unimodal visual stimuli. This test was performed using the apparatus employed for the training. The visual stimuli appeared in one of the 12 spatial locations and lasted 100 ms, with an ISI ranged randomly between 2,000 and 4,000 ms. The test was administered in four sessions, each comprising 100 stimuli: two sessions (200 stimuli) required patients to keep their eyes fixed on the central point (Fixed-Eyes Condition), while in the third and fourth sessions (200 stimuli) they were free to use eye movements to detect visual targets (Eye-Movement Condition), as trained to do during rehabilitation. The camera performed the initial calibration of the position of the patient’s head and eyes during the fixation of the central point. The system suspended the test if the patient did not maintain central point fixation. All 12 spatial positions were exploited: the probability for each possible spatial position was 7%, while for the Catch Trials (condition without visual stimulus) the probability was 16%. The stimuli were distributed randomly in order to verify the reliability of the patient’s responses. The patient was instructed to press the response button to report the detection of visual stimuli.

### Bimodal Audiovisual Test

Visual perception under bimodal audiovisual conditions was also assessed using the apparatus employed for the training procedure. This test quantifies the ability to identify visual stimuli paired with sounds, assessing multisensory integration processes and their benefits for visual orientation. Audiovisual stimuli could appear in different spatial locations to the right and left of the central fixation point. As regards the visual stimuli, each of the 12 LEDs was activated in random order with a probability of 1%, while the acoustic stimuli on the 12 speakers had a probability of 3%. The last condition represents the bimodal audiovisual condition, in which visual stimuli and acoustic stimuli were presented concurrently: the acoustic stimulus could appear in the same position as the visual target with a probability of 1%, or with a nasal/temporal disparity of 16° or 32° with respect to the visual target, with a probability of 1%. For this test, we only used the same spatial positions, from 1 to 5 and from 8 to 12. Central positions (V6–V7) were used only for presenting unimodal stimuli and bimodal spatially congruent stimuli, because in the disparate spatial bimodal condition the acoustic stimulus would appear in the opposite hemifield. Finally, there were also Catch Trials (without visual targets or sounds), presented with a probability of 14%, in a random modality.

The patients were instructed to detect the presence of the visual target by pressing a button and to ignore the auditory stimuli, since they were not predictive of the presence of the visual target. As in the Unimodal test, the test comprised four sessions, of 200 stimuli (visual and acoustic stimuli temporally congruent): two sessions for the Fixed-Eyes Condition and two sessions for the Eye-Movement Condition. The visual stimuli and acoustic targets had the same 100-ms duration. As in the Unimodal test, the ISI ranged randomly between 2,000 and 4,000 ms.

## Preliminary and Anticipated Results

The use of this telerehabilitation system was a positive experience for all three patients and the instrument proved flexible and easy to use at home. In all three subjects, we obtained a good compliance and all of them concluded the training within 5 weeks. They were all able to install the instrument at home on their own, without difficulties. During training only S3 referred some problems, which we discovered were caused by the battery powering the button.

Given the small sample of this preliminary study, results were analyzed separately for each patient. In order to detect differences across multiple behavioural assessments (percentages of responses in visual detection tests), analyses were carried out using non-parametric tests (the Friedman Test and the Wilcoxon test). All analyses were performed using SPSS 20.0 software. For all patients, the percentages of visual detections of stimuli presented in the impaired hemifield were considered.

Both for the Unimodal Visual Test and the Bimodal Audiovisual Test, responses to Catch Trials and to Acoustic Stimuli were not analyzed because the percentages of responses were very low in all tests and for all three patients (<5%).

As expected, no significant differences were found in the computerized visual field perimetry test, before and after the training program.

### Results from S1

#### Unimodal Visual Test

**Figure 4:**
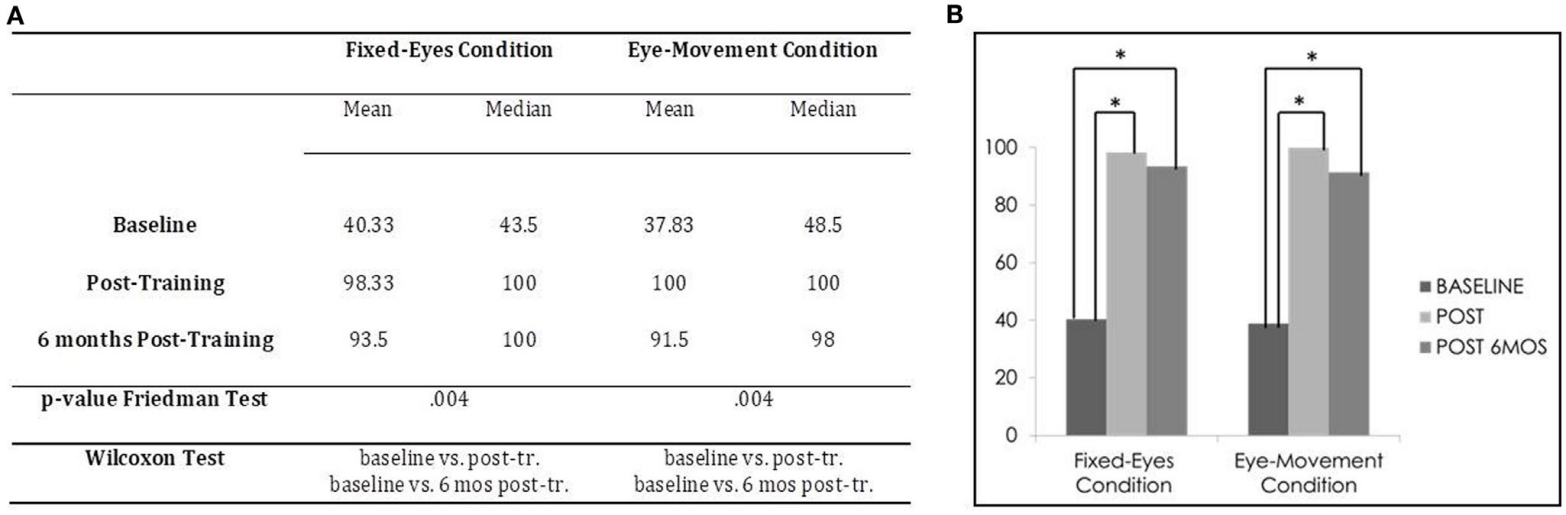
Subject 1: unimodal Visual Test. **(A)** Mean and median percentages of visual detections (hemianopic hemifield) in the different evaluations, *p*-value of the Friedman Tests and results of Wilcoxon Tests. **(B)** Graphical Representation: mean percentages of visual detections (*y*-axis) at baseline vs. post-training vs. 6 months post-training (*x*-axis).

In the Unimodal Visual test, statistical analysis with the Friedman Test showed an important and significant improvement in visual detection rates in the affected hemifield in both the Fixed-Eyes Condition (χ^2^ = 11,200, *p* = 0.004) and in the Eye-Movement Condition (χ^2^ = 11.143, *p* = 0.004). Figure 4 shows the post-training increase of visual detection and the stability of the improvement at the follow-up 6 months after the end of training.

Moreover, post-comparisons with the Wilcoxon Test showed in both the Eye-Movement Condition and Fixed-Eyes conditions a significant difference between the pre-training evaluation and all the other subsequent measures (Fixed-Eyes Condition: baseline vs. post-training, *z* = −2.201, *p* = 0.028, and 6-month follow-up, *z* = −2.201, *p* = 0.028; post-training vs. 6-month follow-up, *z* = −1.1342, *p* = 0.180; Eye-Movement Condition: baseline vs. post-training, *z* = −2.201, *p* = 0.028, and 6-month follow-up, *z* = −2.201, *p* = 0.028; post-training vs. 6-month follow-up, *z* = −1.604, *p* = 0.109) (see Figure 4).

#### Bimodal Audiovisual Test

In the Fixed-Eyes Condition, the percentage of responses to audiovisual stimuli (spatially congruent and incongruent audiovisual stimuli) in the affected hemifield improved appreciably across the different evaluations, as statistically confirmed by the Friedman Test (χ^2^ = 21.385, *p* = 0.0001) (see Table 1). Moreover, the Wilcoxon Test highlighted significant differences between the three measures (baseline vs. post-training, *z* = −3.366, *p* = 0.001, and 6-month follow-up, *z* = −3.123, *p* = 0.002; post-training vs. 6-month follow-up, *z* = −2.207, *p* = 0.027). These results were not confirmed when we analyzed only the responses to audiovisual coincident stimuli across the different evaluations (audiovisual coincident stimuli: χ^2^ = 3.846, *p* = 0.146) and between the three evaluations (all *p* values > 0.1).

**Table 1:** Subject 1: bimodal audiovisual test.

	Fixed-eyes condition				Eye-movement condition			
	Audiovisual stimuli		AV coincident stimuli		Audiovisual stimuli		AV coincident stimuli	
	Mean	Median	Mean	Median	Mean	Median	Mean	Median
Baseline	50.87	58.33	60.71	75	85.16	100	79.92	90.91
Post-training	99.62	100	98.61	100	99.54	100	98.33	100
6 months post-training	91.37	100	92.96	100	93.49	100	97.91	100

*p*-Value Friedman test	0.000		0.146		0.008		0.086	

*Mean and median percentages of visual detections (hemianopic hemifield) in the different evaluations and *p*-value of the Friedman Tests*.

With respect to the Eye-Movement Condition, an improvement in visual detection of audiovisual stimuli (spatially congruent and incongruent audiovisual stimuli) was apparent at the end of the training (χ^2^ = 9.556, *p* = 0.008), with a significant difference between baseline and the post-training assessment (baseline vs. post-training, *z* = −2.668, *p* = 0.008), although a decrease of accuracy was present at 6-month follow-up (baseline vs. 6-month follow-up, *z* = −1,069, *p* = 0.285; post-training vs. 6-month follow-up, *z* = −1.753, *p* = 0.080) (Table 1). These results were not confirmed when we analyzed only the responses to audiovisual coincident stimuli across the different evaluations (Friedman Test, audiovisual coincident stimuli, χ^2^ = 4,906, *p* = 0.086) and between the three evaluations (baseline vs. post-training, *z* = −1,604, *p* = 0.109; baseline vs. 6-month follow-up, *z* = −1.604, *p* = 0.109; post-training vs. 6-month follow-up, *z* = −0.447, *p* = 0.655).

### Results from S2

#### Unimodal Visual Test

**Figure 5:**
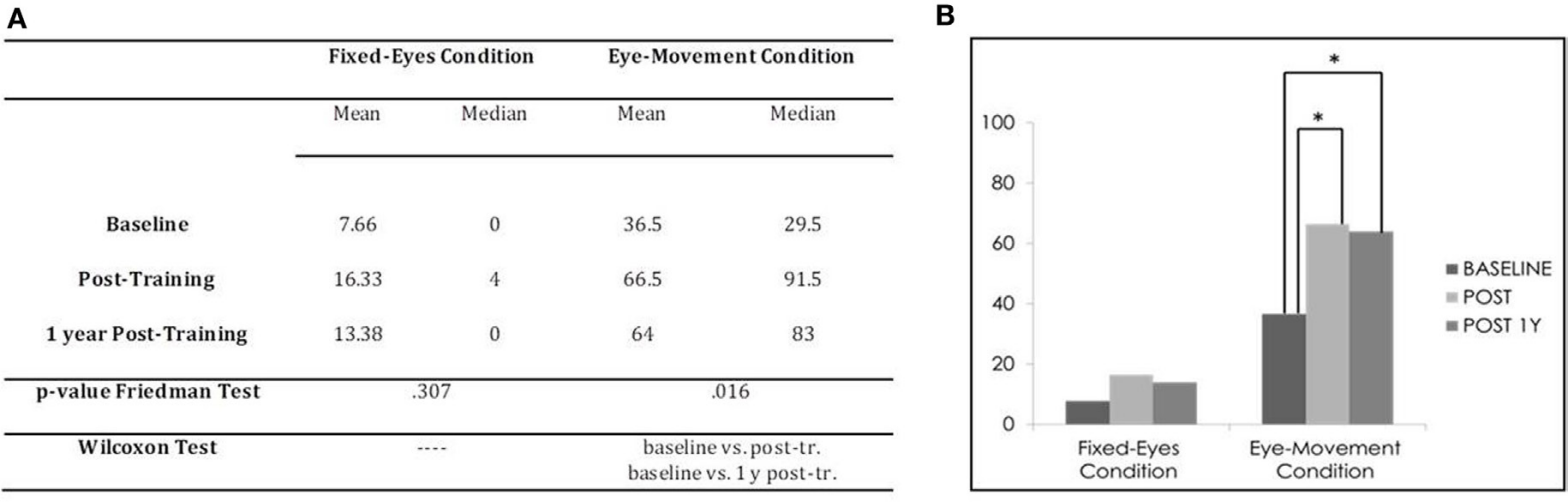
Subject 2: unimodal visual test. **(A)** Mean and median percentages of visual detections (hemianopic hemifield) in the different evaluations, *p*-value of the Friedman Tests and results of Wilcoxon Tests. **(B)** Graphical Representation: mean percentages of visual detections (*y*-axis) at baseline vs. post-training vs. 1 year post-training (*x*-axis).

In the Unimodal Visual test, statistical analysis with the Friedman Test showed significant differences in visual detection rates in the affected hemifield in the Eye-Movement Condition (χ^2^ = 8.316, *p* = 0.016), while no significant difference was found in the Fixed-Eyes Condition (χ^2^ = 2.364, *p* = 0.307) (see Figure 5).

Post comparison with the Wilcoxon Test showed significant differences between the pre-training evaluation and all other subsequent measures in the Eye-Movement Condition (baseline vs. post-training, *z* = −2.2013, *p* = 0.043, and 1 year follow-up, *z* = −2.203, *p* = 0.043; post-training vs. 1 year follow-up, *z* = −0.730, *p* = 0.465), while no difference was found in the Fixed-Eyes Condition (all *p* values > 0.1) (see Figure 5).

#### Bimodal Audiovisual Test

In the Fixed-Eyes Condition, the percentage of responses to audiovisual stimuli (spatially congruent and incongruent audiovisual stimuli) and the responses to audiovisual coincident stimuli in the affected hemifield did not improve across the different evaluations (Friedman Test, audiovisual stimuli, χ^2^ = 0.800, *p* = 0.670; Friedman Test, audiovisual coincident stimuli, χ^2^ = 2.000, *p* = 0.368).

Instead, in the Eye-Movement Condition, the Friedman Test and the Wilcoxon Test highlighted a significant change in the visual detection of audiovisual stimuli (spatially congruent and incongruent audiovisual stimuli) across measures (χ^2^ = 14.993, *p* = 0.001) and between measures (baseline vs. post-training, *z* = −2.905, *p* = 0.004; baseline vs. 1 year follow-up, *z* = −2.803, *p* = 0.005; post-training vs. 1 year follow-up, *z* = −209, *p* = 0.037), but the improvement in responses to audiovisual coincident stimuli were not confirmed (Friedman Test, audiovisual coincident stimuli, χ^2^ = 3.500, *p* = 0.174; Wilcoxon Test, baseline vs. post-training, *z* = −1.095, *p* = 0.273; baseline vs. 1 year follow-up, *z* = −1.826, *p* = 0.068; post-training vs. 1 year follow-up, *z* = −0.730, *p* = 0.465) (see Table 2).

**Table 2:** Subject 2: bimodal audiovisual test.

	Fixed-eyes condition				Eye-movement condition			
	Audiovisual stimuli		AV coincident stimuli		Audiovisual stimuli		AV coincident stimuli	
	Mean	Median	Mean	Median	Mean	Median	Mean	Median
Baseline	1.58	0	3.03	0	52.64	50	55.53	61.60
Post-training	4.54	0	16.67	0	83.79	100	75.56	95.45
1 year post-training	5.55	0	20.36	0	68.79	100	65.03	80.81

*p*-Value Friedman test	0.670		0.368		0.001		0.174	

*Mean and median percentages of visual detections (hemianopic hemifield) in the different evaluations and *p*-value of the Friedman Tests*.

### Results from S3

#### Unimodal Visual Test

**Figure 6:**
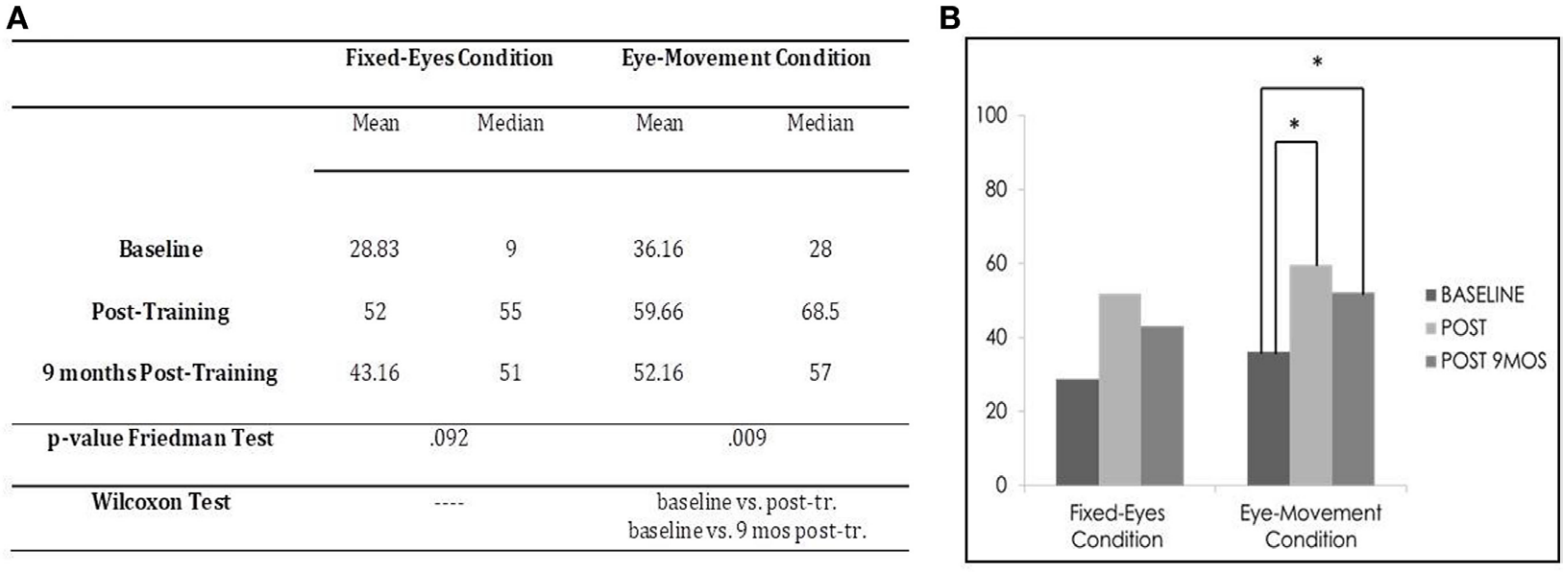
Subject 3: unimodal visual test. **(A)** Mean and median percentages of visual detections (hemianopic hemifield) in the different evaluations, *p*-value of the Friedman Tests and results of Wilcoxon Tests. **(B)** Graphical Representation: mean percentages of visual detections (*y*-axis) at baseline vs. post-training vs. 9 months post-training (*x*-axis).

In the Unimodal Visual test, statistical analysis with the Friedman Test showed a significant difference in visual detection rates in the affected hemifield in the Eye-Movement Condition (χ^2^ = 9.364, *p* = 0.009), while no significant difference was found in the Fixed-Eyes Condition (χ^2^ = 4.778, *p* = 0.092). Figure 6 shows the post-training increase until 9 months after the training.

Moreover, in the Eye-Movement Condition, post comparison with the Wilcoxon Test highlighted a significant difference between the pre-training evaluation and all the other subsequent measures (Eye-Movement Condition, baseline vs. post-training, *z* = −2.201, *p* = 0.028, and 9-month follow-up, *z* = −2.032, *p* = 0.042; post-training vs. 9-month follow-up, *z* = −1.625, *p* = 0.104), while in the Fixed-Eyes Condition, the same improvement was not detected, even if there seemed to be a slight amelioration of percentage after training (tending to statistical significance), but this was not confirmed at 9-month follow-up (baseline vs. post-training, *z* = −1.826, *p* = 0.068, and vs. 9-month follow-up, *z* = −1.461, *p* = 0.144; post-training vs. 9-month follow-up, *z* = −0.944, *p* = 0.345) (see Figure 6).

#### Bimodal Audiovisual Test

In the Fixed-Eyes Condition, the percentage of responses to audiovisual stimuli (spatially congruent and incongruent audiovisual stimuli) in the hemianopic hemifield improved appreciably across the different evaluations and the Friedman Test confirmed this significant change (audiovisual stimuli: χ^2^ = 21.562, *p* = 0.00001). Also, the Wilcoxon Test underlined significant differences between the three measures (baseline vs. post-training, *z* = −3.824, *p* = 0.00001; and vs. 9-month follow-up, *z* = −2.225, *p* = 0.026; post-training vs. 9-month follow-up, *z* = −2.287, *p* = 0.020). These results were not confirmed when we analyzed only the responses to audiovisual coincident stimuli across the different evaluations (audiovisual coincident stimuli, χ^2^ = 5.158, *p* = 0.076) and between the three evaluations (baseline vs. post-training, *z* = −1.826, *p* = 0.068; and 9-month follow-up, *z* = −0.674, *p* = 0.500; post-training vs. 9-month follow-up, *z* = −1.225, *p* = 0.225).

Similar results were obtained in the Eye-Movement Condition. In fact, statistical analysis showed an improvement in the visual detection of audiovisual stimuli (spatially congruent and incongruent audiovisual stimuli) across the evaluations (Friedman Test, audiovisual stimuli, χ^2^ = 26.132, *p* = 0.00001) and between the three evaluations (Wilcoxon Test, baseline vs. post-training, *z* = −3.926, *p* = 0.00001, and vs. 9-month follow-up, *z* = −2.677, *p* = 0.007; post-training vs. 9-month follow-up, *z* = −2.487, *p* = 0.013). Also in this condition, the responses to audiovisual coincident stimuli were significant; however, the comparison between baseline and follow-up did not show significant differences (Friedman Test, audiovisual coincident stimuli, χ^2^ 9.478, *p* = 0.009; Wilcoxon Test, baseline vs. post-training, *z* = −2.207, *p* = 0.027; and vs. 9-month follow-up, *z* = −0.405, *p* = 0.686; post-training vs. 9-month follow-up, *z* = −2.207, *p* = 0.027) (see Table 3).

**Table 3:** Subject 3: bimodal audiovisual test.

	Fixed-eyes condition				Eye-movement condition			
	Audiovisual stimuli		AV coincident stimuli		Audiovisual stimuli		AV coincident stimuli	
	Mean	Median	Mean	Median	Mean	Median	Mean	Median
Baseline	25.18	0	33.81	21.43	42.58	50	49.46	63.88
Post-training	67.06	78.89	56.75	62.5	84.42	100	72.05	92.85
9 months post-training	48.93	64.58	38.20	40	65.99	77.5	47.11	58.57

*p*-Value Friedman test	0.000		0.076		0.000		0.009	

*Mean and median percentages of visual detections (hemianopic hemifield) in the different evaluations and *p*-value of the Friedman Tests*.

### Notes

To ensure that the training program gives optimal results the subject should practice every day. For this reason, patients are controlled remotely by medical staff. A possible risk with this procedure is that the subject could choose the wrong exercise for the day; however, it is possible to verify remotely which type of exercise is being done and correct the error in real time.

## Discussion

The incidence of visual field defects is expected to increase due to the improvement in health care and the increase in patients’ life span. This type of sensory disorder can be very debilitating and interfere with daily life: besides the negative impact on stroke prognosis, loss of independence and inability to enjoy leisure activities are likely to have significant negative emotional and social implications. Since complete spontaneous recovery is rare, the search for methods that reduce these disabilities becomes indispensable. The results of the present study, albeit with a limited number of patients, suggest the therapeutic value of a telerehabilitation system based on the multisensory stimulation of the visual field to promote the development of oculomotor compensatory abilities in order to overcome the visual field loss.

Evidence in adult patients with chronic visual field defects following a stroke has proved the efficacy of audiovisual training in reinforcing visual and spatial compensatory functions. Animal models have also shown that auditory-visual training reinstates visuomotor competencies in animals rendered hemianopic by complete unilateral visual cortex ablation. Such a recovery of visual behaviors was shown to be linked to reinstatement of visual responsiveness in deep layer multisensory neurons of the superior colliculus (34), suggesting that multisensory stimulation training can guide brain plasticity after the lesion, facilitating the recovery of visual orientation skills. In fact, as reported in previous research (18–20, 35), the results of the present study confirm the effectiveness of treatments based on the stimulation of ocular movements and visual exploration functions through compensative strategies. This amelioration was observable primarily in the Eye-Movement Condition, because patients were instructed to use saccadic eye movements for the detection of visual targets and thus they showed, at the end of the treatment, an activation of the oculomotor system and a change in responsiveness toward visual stimuli, confirmed by behavioral data, mostly using the Unimodal Visual Test. Bolognini and colleagues (18) have suggested that the discrepancy of results between the Fixed-Eyes Condition and Eye- Movement Condition suggests that the improvement in visual perception induced by the training intervention is not due to an enlargement of the visual field, but it is mostly mediated by the oculomotor system.

Here for the first time, we provide evidence that a home-based telerehabilitation system for audiovisual training could be a valuable addition to standard treatment protocols. An optimal rehabilitation program should be effective, simple to use, portable, and low-cost, and our approach seems to meet these criteria. In fact, telerehabilitation could have a bright future in the recovery of neurological and neuropsychological functions, since it is cost-effective and is accessible to more patients, relieving current pressure on the health-care system, which is challenged by an aging society and by an increase in survival rates after brain injuries. Our new therapy for visual field defects allows patients to exercise independently, in an intensive and active way and, above all, in a familiar context, while under remote supervision. It gives the patient a sense of control and autonomy, which can contribute to a better therapy outcome, also reducing the need for one-to-one treatment time and home visits. Telerehabilitation also enables prolonged training for patients who may be unable to access it, due to resource and service restrictions within the health-care system. To our knowledge, this is the first attempt to use telerehabilitation with adults with chronic visual field defects and we showed how this approach proved itself useful for the first three patients who tried it. This is an important result since Bittner and colleagues (36) affirm in a recent review that they “did not find any evidence on whether the use of telerehabilitation is feasible or a potentially viable means to remotely deliver rehabilitation services to individuals with low vision.”

Now, our aim is to test these preliminary findings in a larger sample, and to evaluate the efficacy and feasibility of the AVT system in both adults and children with visual field defects.

## Ethics Statement

The study was carried out in accordance with the recommendations of the Scientific Institute “Stella Maris Foundation” institutional review board. Patients signed an Informed Consent Form, in accordance with the Declaration of Helsinki, authorizing the storage and study of their clinical material for research aims.

## Author Contributions

FT, GC, and GP participated in the design, execution, and data analysis of the paper entitled “Development and implementation of a new telerehabilitation system for Audiovisual Stimulation Training in Hemianopia” and declare they have seen and approved the final version and that it has neither been published nor submitted elsewhere.

## Conflict of Interest Statement

The authors declare that the research was conducted in the absence of any commercial or financial relationships that could be construed as a potential conflict of interest. The reviewer RG and handling editor declared their shared affiliation.
